# Clinically applicable antibiotic modification of ready-to-use calcium phosphate cement pastes

**DOI:** 10.1186/s13018-025-06217-w

**Published:** 2025-08-22

**Authors:** Alicia Zeiner, Johannes K. Konrad, Sandra Hinderer, Andreas Hoess, Sascha Heinemann, Uwe Gbureck

**Affiliations:** 1 Department for Functional Materials in Medicine and Dentistry, University Hospital Würzburg, Pleicherwall 2, 97070 Würzburg, Germany; 2 Department for Orthodontics, University Hospital Würzburg, Pleicherwall 2, 97070 Würzburg, Germany; 3 INNOTERE GmbH, Meissner Strasse 191, 01445 Radebeul, Germany

**Keywords:** Calcium phosphate, Bone cement, Antibiotics, Ready-to-use, Paste

## Abstract

**Background:**

Ready-to-use non-aqueous cement pastes consist of an organic, water-miscible liquid phase in which cement powder is dispersed. While the modification of classical aqueous powder/liquid cement pastes with antibiotics has been extensively investigated, only a few studies previously published aimed at drug modification of premixed cement pastes.

**Methods:**

A simple method for modifying such ready-to-use pastes with the model antibiotic vancomycin is described. It was demonstrated that mixing the cement paste and vancomycin solution multiple times using an adapter between two syringes resulted in macroscopically homogeneous pastes. The antibiotic-loaded cements were thoroughly characterized with regard to their physicochemical properties and their drug release profile.

**Results:**

Drug modification with vancomycin had no adverse effects on cement properties in terms of mechanical performance, phase composition, porosity and pore size distribution. Drug release from ready-to-use pastes exhibited a lower initial burst release of 14.5% compared to 47.9% for aqueous powder/liquid cement pastes after 24 h. At later time points, the initial exponential release kinetics slowed down due to both a decreased concentration gradient and structural changes of the cement matrix during setting. This resulted in sustained release of the antibiotic for more than 30 days. The released vancomycin maintained high antimicrobial activity against S. aureus with inhibition zones of 5.4–6.8 mm in an agar diffusion test.

**Conclusion:**

The results demonstrated that modification of ready-to-use cement pastes with vancomycin is a potential candidate for clinical application, as it preserves both the material’s properties and the antibiotic’s activity.

## Background

The therapy of an infection in the bone (e.g. osteomyelitis) often poses great challenges for successful treatment, as the bone is difficult to access for many antibiotics due to its dense structure and poor blood supply to the cortical bone. This often requires high systemic drug concentrations as well as a long period of treatment for usually a couple of weeks [1–3]. The high doses systemically administered also increase the probability for the occurrence of side effects and toxicity. In multimorbid patients, this results in longer hospitalization and sometimes permanent health impairments with correspondingly high costs for healthcare and social systems. In order to circumvent the aforementioned problems, an intraoperative local antibiotic therapy is an important alternative to avoid high systemic doses and to achieve a locally sufficient concentration of the active substance [3]. Hence, nowadays the therapy of osteomyelitis of long bones often includes the combination of surgical rehabilitation with local antibiotic therapy [4]. For clinical use, various forms of defect fillers modified with antibiotics already exist, usually consisting of polymethyl methacrylate (PMMA) in the form of chains or spheres with added vancomycin, gentamicin, and rifampicin [5]. Likewise, some self-setting PMMA cements are also available with either admixtures of antibiotics or these drugs can be added to the cement intraoperatively [6]. Treatment options for infected implants involve removing the implant, followed by a temporary implantation of drug-loaded spacers made from PMMA cement. However, PMMA cements release heat during the setting reaction and even more importantly they are not resorbable and have to be surgically removed after treatment. Calcium phosphate cements (CPC) are an alternative, especially in non-load-bearing skeletal sections as they set at ambient temperature and can be replaced by newly formed bone after cement degradation. However, CPC are currently not available with admixed antibiotics, and manual admixture of the active substances is usually not approved and can therefore only be performed off-label. This requires precise knowledge of the release kinetics and the pharmacological efficacy of the antibiotic must not be impaired by the cement [7]. It must also be excluded that changes in the properties of the cement (e.g. mechanical strength, composition) occur due to the modification with antibiotics. While these factors have already been investigated for a variety of aqueous powder/liquid calcium phosphate cements in combination with a whole range of antibiotics for both in vitro [8] and clinical studies [9–11], the modification of the relatively new class of ready-to-use non-aqueous cement pastes with antibiotics has been the subject of very few studies. This class of cement consists of an organic, water-miscible liquid phase in which the cement powder is dispersed [12–14]. Such pastes are storage-stable and only harden after injection into the moist environment of a bone defect due to the diffusion of water into the paste matrix initiating the cement reaction. This procedure has decisive clinical advantages, as there is no susceptibility to errors during the mixing process and unlimited time for application. The applicability of prefabricated ready-to-use calcium phosphate cement pastes has been demonstrated in various ex vivo, in vivo, and clinical studies [15–18]. However, when modifying such ready-to-use pastes with active ingredients, the practical question arises of how the clinical user can homogeneously add the drug to the paste. Vorndran et al. [19] described two approaches in which the active ingredient was either mixed as a powder concentrate with the solid cement phase or dissolved in the liquid phase and subsequently combined using a static mixing unit during injection.

In this work, an additional method for homogeneous mixing is introduced, which does not require the user to carry out weighing of cement and drug powders. This makes the method readily applicable in clinical settings, as it can be integrated into routine workflows with minimal effort and low risk of user error. Furthermore, both the drug content and the volume of liquid used can be easily and specifically adjusted to meet the respective requirements. The objective of this work was, therefore, to investigate the manual admixture of vancomycin as a model antibiotic to a ready-to-use, oil-based cement paste [12]. The admixture was carried out by connecting two syringes filled with active ingredient solution or cement paste via an adapter and then injecting the syringe contents back and forth several times. The aim was to identify possible influencing factors on the release kinetics of the injected active ingredient. Therefore, the amount and concentration of the active ingredient solution were varied and the influence of these modifications on the release, the mechanical properties, the porosity and the composition of the hardened cement bodies was investigated. In addition, reference measurements were also compared with a cement of the same composition from an aqueous powder/liquid system.

## Materials and methods

The ready-to-use cement paste, commercially available as the medical device INNOTERE Paste-CPC, was provided by INNOTERE GmbH (Radebeul, Germany) and prepared as described previously [9]. In brief, a precursor powder comprising α-tricalcium phosphate (60 wt%), dicalcium phosphate anhydrous (monetite, 26 wt%), precipitated hydroxyapatite (4 wt%) and calcium carbonate (calcite, 10 wt%) was mixed with dipotassium hydrogen phosphate (2.5 wt%) and dispersed in an oil-based carrier liquid consisting of Myglyol 812, castor oil ethoxylate 35 (14.7 wt%) and hexadecyl phosphate (4.9 wt%) using a stainless steel mixer to form a pasty calcium phosphate cement.

### Cement modification with vancomycin

**Fig. 1:**
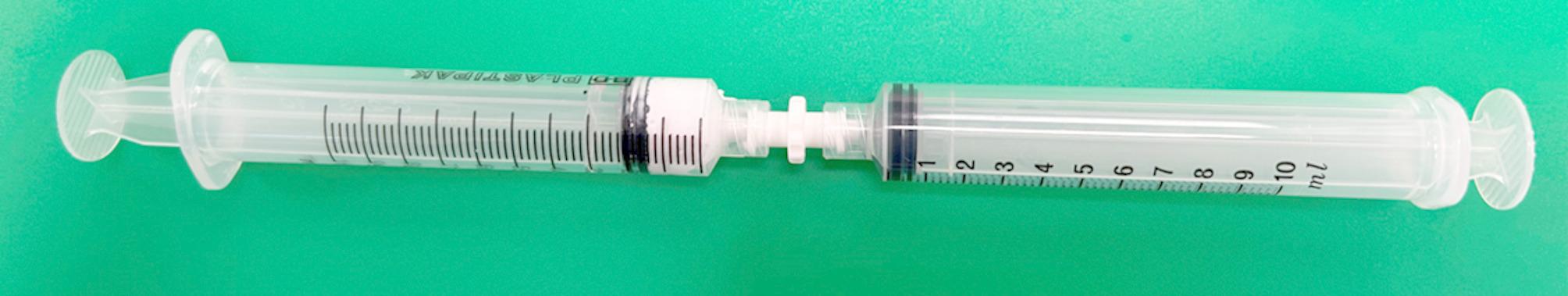
Setup for manually mixing ready-to-use cement paste with vancomycin solution

Mixing of the cement paste with vancomycin hydrochloride (Demo Pharmaceuticals, Hallbergmoos, Germany) solution containing either 30 mg, 40 mg or 60 mg of the drug was carried out by connecting two syringes with a Luer-Lock adapter (Masterflex, VWR, Germany), whereas one syringe contained 3.7 g of the cement paste and the second syringe was filled with either water (as reference) or 0.6–1 ml vancomycin solution (Fig. 1). The mixing process started by fully injecting the cement paste into the aqueous phase, followed by complete reinjection of the mixture into the starting syringe. This process was repeated ten times within a time window of 1 min. The solutions used and the resulting vancomycin concentration in the cement samples are shown in Table 1. The vancomycin content of each sample was calculated using the weight of the samples. A water-based reference cement (powder/liquid) was fabricated by mixing the pure powder phase (INNOTERE, Radebeul, Germany) with a 2.5 wt% solution of disodium hydrogen phosphate containing 24.1 mg/ml vancomycin at a powder to liquid ratio of 2.5 g/ml.

**Table 1:** Vancomycin concentrations of the solutions used for the fabrication of the drug-loaded samples from 3.7 g ready-to-use cement paste in mg/ml as well as the resulting vancomycin concentration in the cement samples in mg/g

Sample denomination	Total amount of vancomycin in solution	Amount and concentration of vancomycin solution	Vancomycin in set cement
30 mg / 0.6 ml	30 mg	0.6 ml of 50.0 mg/ml	6.98 mg/g
30 mg / 0.8 ml		0.8 ml of 40.0 mg/ml	6.67 mg/g
30 mg / 1.0 ml		1.0 ml of 30.0 mg/ml	6.35 mg/g
40 mg / 0.6 ml	40 mg	0.6 ml of 66.7 mg/ml	9.30 mg/g
40 mg / 0.8 ml		0.8 ml of 53.4 mg/ml	8.89 mg/g
40 mg / 1.0 ml		1.0 ml of 40.0 mg/ml	8.51 mg/g
60 mg / 0.6 ml	60 mg	0.6 ml of 100.0 mg/ml	13.95 mg/g
60 mg / 0.8 ml		0.8 ml of 80.0 mg/ml	13.33 mg/g
60 mg / 1.0 ml		1.0 ml of 60.0 mg/ml	12.77 mg/g

All cement pastes were transferred into cuboid silicon rubber molds with dimensions of 12 × 6 × 6 mm³ and set for 1 h at 37 °C and 100% relative humidity for the release experiments. Samples for mechanical testing, porosity measurements, and phase analysis were stored under the same conditions for 7 days.

### Vancomycin release

After mixing and storage for one hour as described above, the samples were immersed in 5 ml phosphate-buffered saline (PBS) solution each and stored at 37 °C on a shaker. The medium was changed after 30 min, 1, 2, 3, 4, 6, 8 h, then once a day until day 14, and every other day until day 34. The released vancomycin was quantified by UV-Vis spectroscopy (Genesys 10 S UV-Vis, Thermo Fisher Scientific, Waltham, USA) at 280 nm using a calibration curve with vancomycin concentrations in the range of 0.1–10 mg/l. After finishing the release experiment, the samples were dissolved in 15 ml of 32% hydrochloric acid and the remaining vancomycin was determined accordingly by UV-Vis spectroscopy.

### Cement analysis

The initial setting of the pastes after mixing with water or vancomycin solution was measured according to the Gillmore needle test with a needle diameter of 2.12 mm and a weight of 113.4 g at 37 °C and a humidity of > 90%. The determination of the compressive strength was carried out on a universal testing machine Z010 (ZwickRoell, Ulm, Germany) with a 10 kN load cell at a speed of 1 mm/min (*n* = 5). The software testXpert II (ZwickRoell, Ulm, Germany) was used for the calculation of the compressive strengths. The composition of the samples (*n* = 3) was determined using the X-ray diffractometer D8 Advance DaVinci (Bruker, Billerica, USA). The samples were ground in a mortar and placed on the sample plate. The measurement was carried out at 2ϴ angles between 7° and 70° at a step speed of 0.4 s/step and a step size of 0.02°. The qualitative composition was determined using DIFFRAC.EVA software (Bruker Corporation, Billerica, USA). The quantitative composition of the samples was evaluated by means of Rietveld analysis with the software DIFFRAC.TOPAS (Bruker, Billerica, USA). The porosity of the hardened cements (*n* = 3) was analyzed by mercury porosimetry (Hg porosimetry). The measurement was performed using a PASCAL 140/440 mercury porosimeter (Thermo Fisher Scientific, Waltham, USA) with the associated SOLiD^®^ evaluation software (Thermo Fisher Scientific, Waltham, USA). All measurements were taken at a mercury surface tension of 0.48 N/m and a contact angle of 140°.

### Antimicrobial activity of released vancomycin

Lysogeny broth (LB) medium was prepared from 2 g yeast extract (AppliChem, Darmstadt, Germany), 4 g tryptone (AppliChem, Darmstadt, Germany), and 2 g sodium chloride (Sigma-Aldrich, St. Louis, USA) in 400 ml ultrapure water. For the overnight culture, a small amount of an S. aureus culture (ATCC 6538) stored at -80 °C in glycerol was scraped off with a pipette tip and suspended in 10 ml LB medium. The culture was stored for 12 h at 225 rpm on a laboratory shaker with an incubator (Unimax 1010 and Incubator 1000, Heidolph Instruments, Schwabach, Germany) at 37 °C. Subsequently, two agar plates were inoculated using a three-eye spread and incubated at 37 °C (Kelvitron^®^ T, Thermo Fisher Scientific, Waltham, USA) for 24 h. For the inhibition test, 100 µL of the overnight culture from the single colony was added to the centre of an agar plate and evenly distributed using approximately 20 glass beads. The test specimens were placed on the agar plate with tweezers and then stored in the incubator at 37 °C for 24 h. Subsequently, the inhibition zone size was measured at six different locations by means of a calliper.

### Statistics

The statistical evaluation and data analysis of the results were carried out with OriginPro 2020 (OriginLab, Northampton, USA). The mean value and standard deviation were determined for the statistical evaluation. From these, significance was assessed using a one-way ANOVA for independent variables, following confirmation of normality using the Shapiro-Wilk test, depending on the structure of the data sets (SigmaPlot 12.5 software, Grafiti LLC, Palo Alto, USA). All deviations were considered to be statistically significant with a p-value of *p* ≤ 0.05.

## Results

**Fig. 2:**
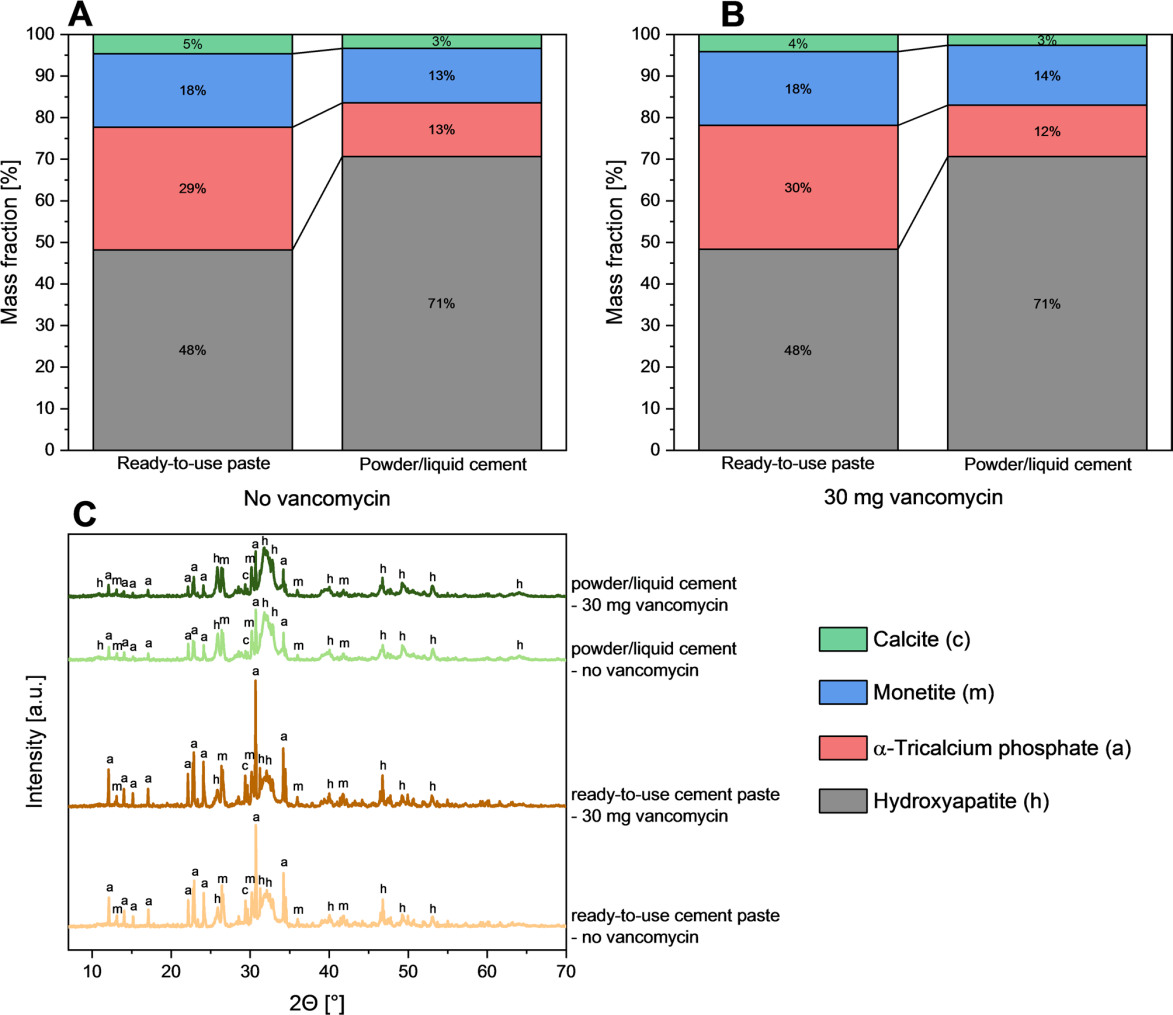
Quantitative phase composition of ready-to-use and powder/liquid cement pastes after 7 days at 37 °C in PBS, both **(A)** without or **(B)** with (6.98 mg/g cement) drug loading, as well as **(C)** the corresponding underlying X-ray diffractograms

After 7 days of setting, noticeable differences in the mineral phase composition were observed between the powder/liquid cement and the cement from the ready-to-use paste, whereas the addition of a small amount of vancomycin (6.98 mg/g of cement) had only a minimal effect (Fig. 2). The powder/liquid cements achieved a conversion into hydroxyapatite as the final cement setting product of approximately 71%, while the ready-to-use pastes exhibited a hydroxyapatite content of 48%, irrespective of whether vancomycin was added or not.

**Fig. 3:**
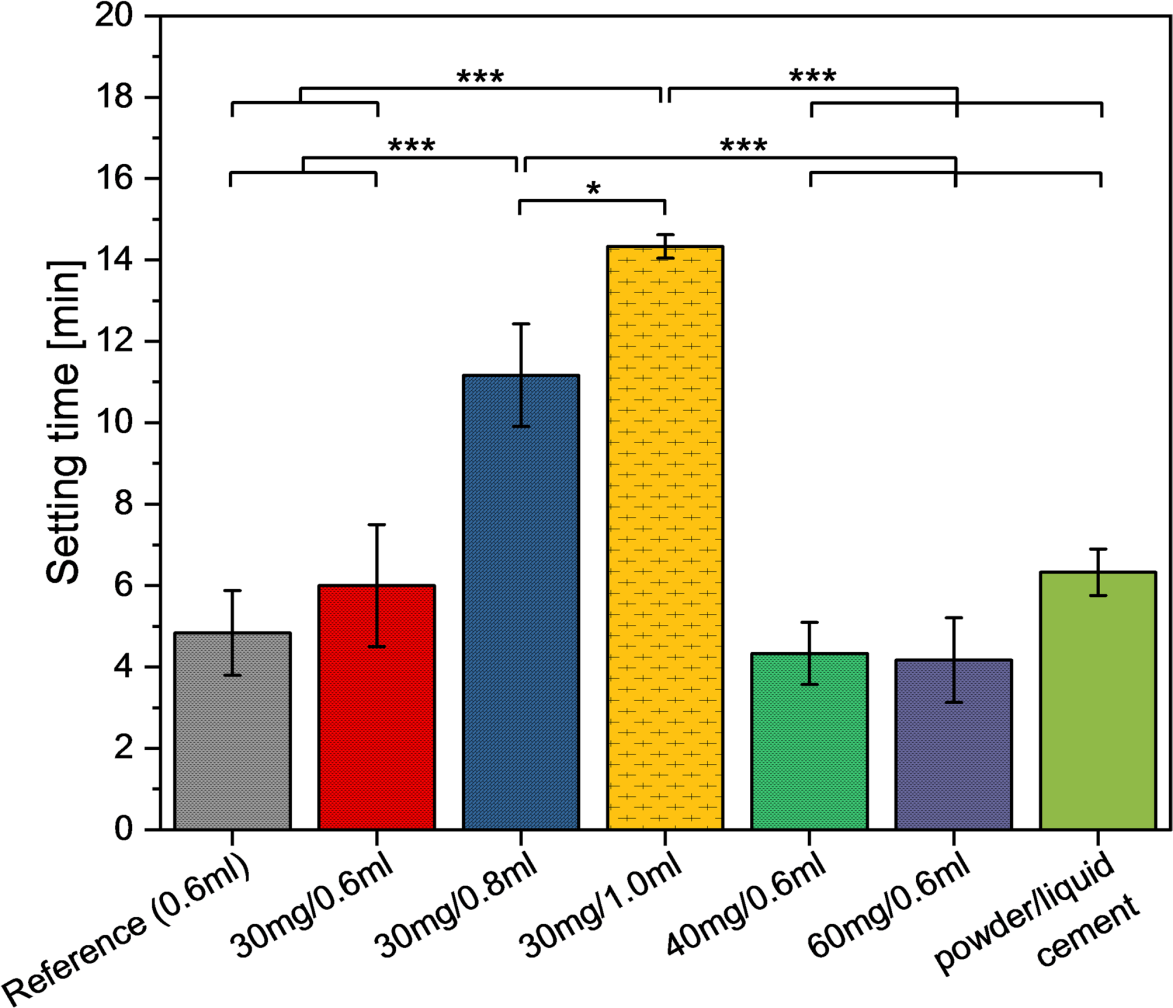
Setting times of ready-to-use cement paste mixed with water (reference) or vancomycin solution with 30, 40, and 60 mg in 0.6–1.0 ml and setting time of powder/liquid cement. (*p < 0.05, ***p < 0.001)

In further experiments, we investigated the effect of drug loading on the setting behaviour and mechanical performance of the different cement mixtures. Modification of the ready-to-use cement paste with an aqueous antibiotic solution initiated the setting process. Pure water (0.6 ml) used as a reference resulted in an initial setting time of 4.8 ± 1.0 min according to the Gillmore needle test. Adding 30–60 mg vancomycin to the 0.6 ml of water did not significantly change the setting time, whereas a higher amount of solution increased the setting time significantly to 11.3 ± 1.3 min (0.8 ml) and 14.3 ± 0.3 min (1.0 ml) (Fig. 3). The setting time of an aqueous powder/liquid cement with the same cement composition was 6.3 ± 0.6 min.

**Fig. 4:**
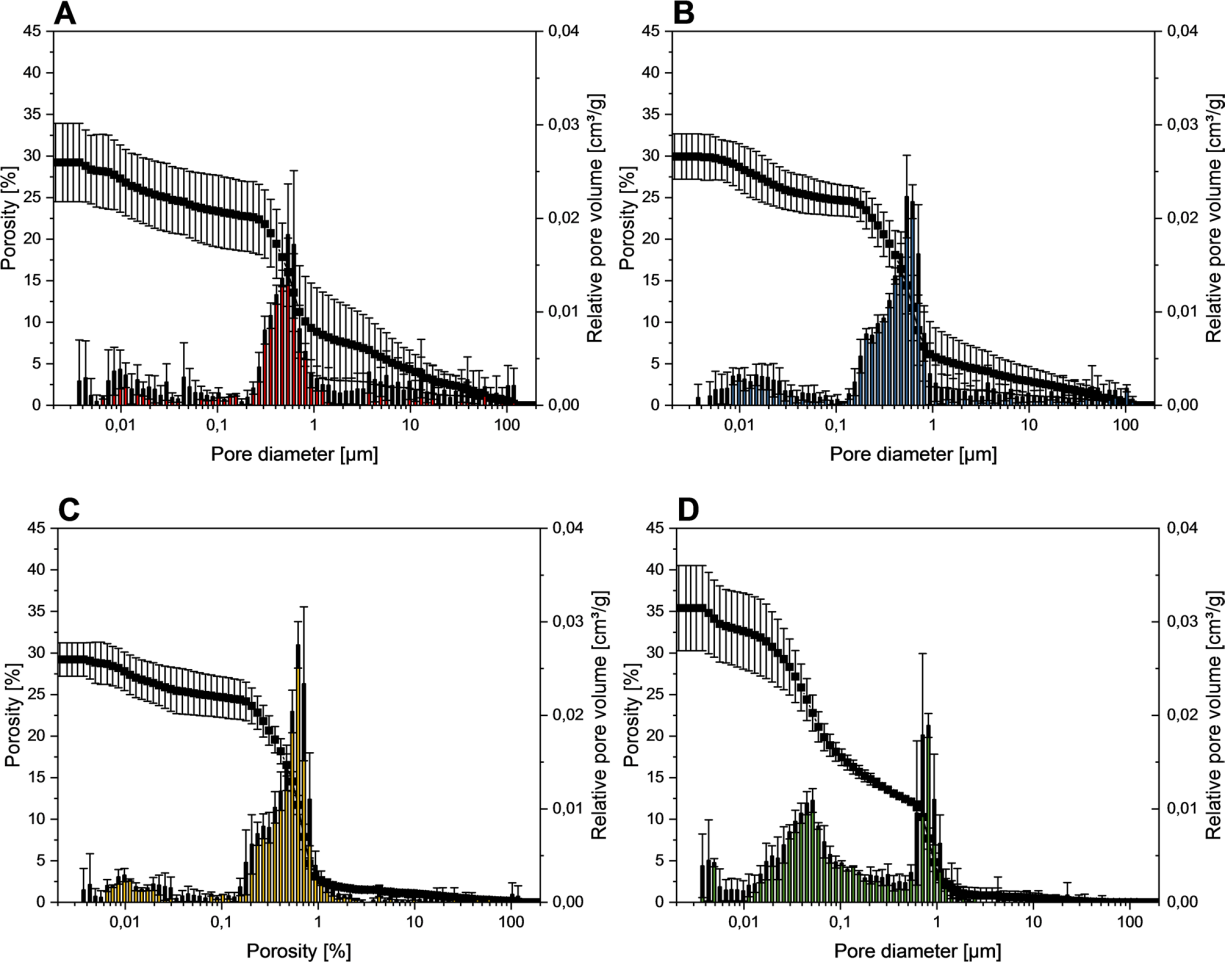
Porosity and pore size distribution of cements modified with different volumes of vancomycin solution (total amount 30 mg) after 7 d setting at 37 °C in PBS. **A**-**C**: Ready-to-use pastes (3.7 g) prepared with (**A)** 0.6 ml solution (6.98 mg/g vancomycin), (**B)** 0.8 ml solution (6.66 mg/g vancomycin), and (**C)** 1.0 ml solution (6.35 mg/g vancomycin). **D**: Powder/liquid cement (6.98 mg/g vancomycin)

According to the International Union of Pure and Applied Chemistry (IUPAC), the antibiotic-loaded cements were found to be microporous, as shown by mercury porosimetry (Fig. 4) [20]. Ready-to-use pastes had a total porosity of approximately 28–30% with a bimodal pore size distribution, showing one peak between 10 and 50 nm corresponding to mesopores, and a more pronounced peak between 500 and 800 nm corresponding to macropores. In contrast, the powder/liquid cements provided a higher total porosity (35%) as well as a bimodal pore size distribution, with two maxima around 50 nm and 800 nm.

**Fig. 5:**
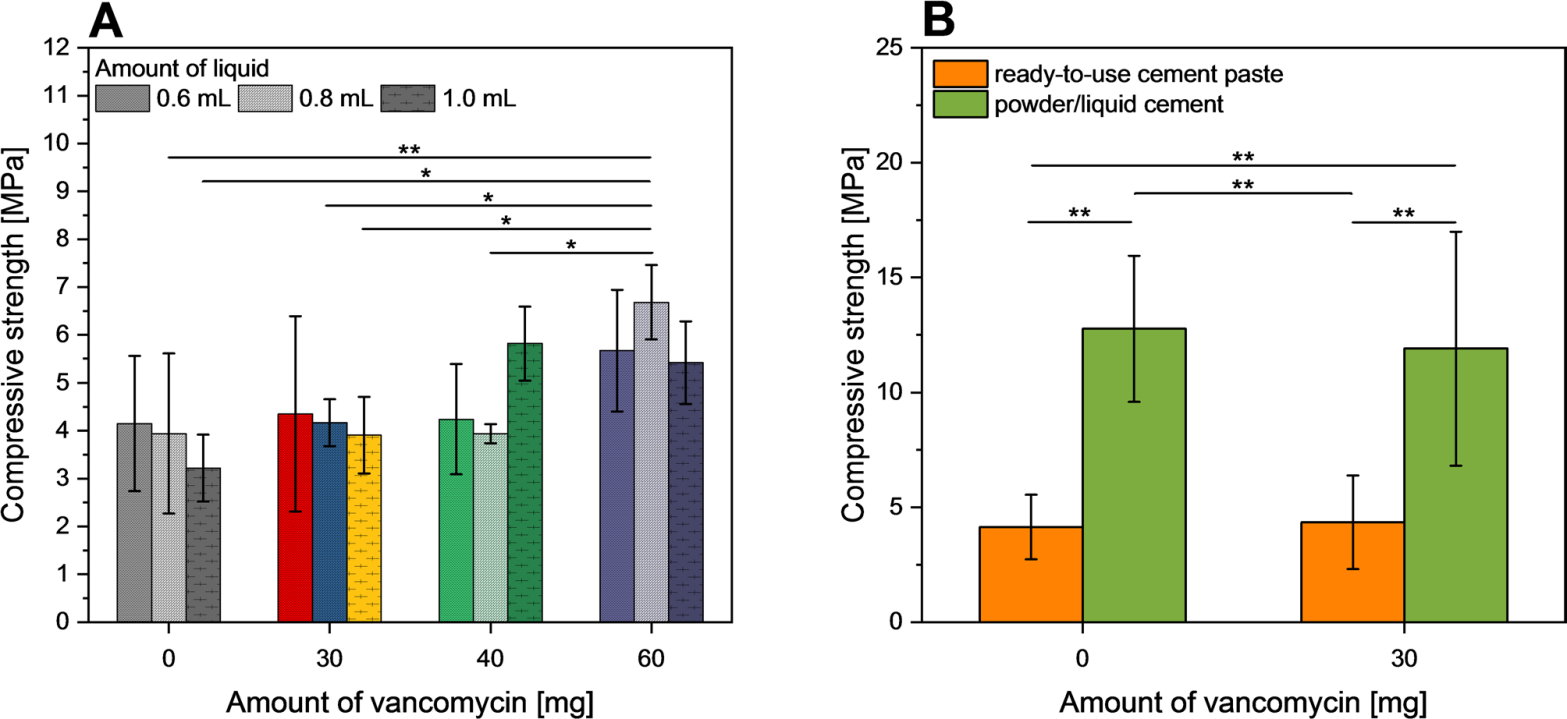
Compressive strength of **(A)** cement samples obtained by mixing 3.7 g ready-to-use cement paste with different amounts of liquid containing 0, 30, 40, or 60 mg vancomycin; cements were hardened for 7 d at 37 °C in PBS. **(B)** Comparison of ready-to-use vs. powder/liquid cement pastes without or with (6.98 mg/g cement) drug loading. (*p < 0.05, **p < 0.01)

While lower amounts of vancomycin (30 and 40 mg) had practically no effect on the compressive strength of the cements after 7 days of hardening, the addition of 60 mg of the antibiotic slightly improved the mechanical performance (Fig. 5A). When comparing powder/liquid cements and ready-to-use pastes with and without drug loading (Fig. 5B), the powder/liquid cements showed compressive strengths approximately three times higher. However, for both cement systems, there was no observed effect on the mechanical performance of the samples containing 30 mg vancomycin (= 6,98 mg/g) compared to the antibiotic-free cements.

**Fig. 6:**
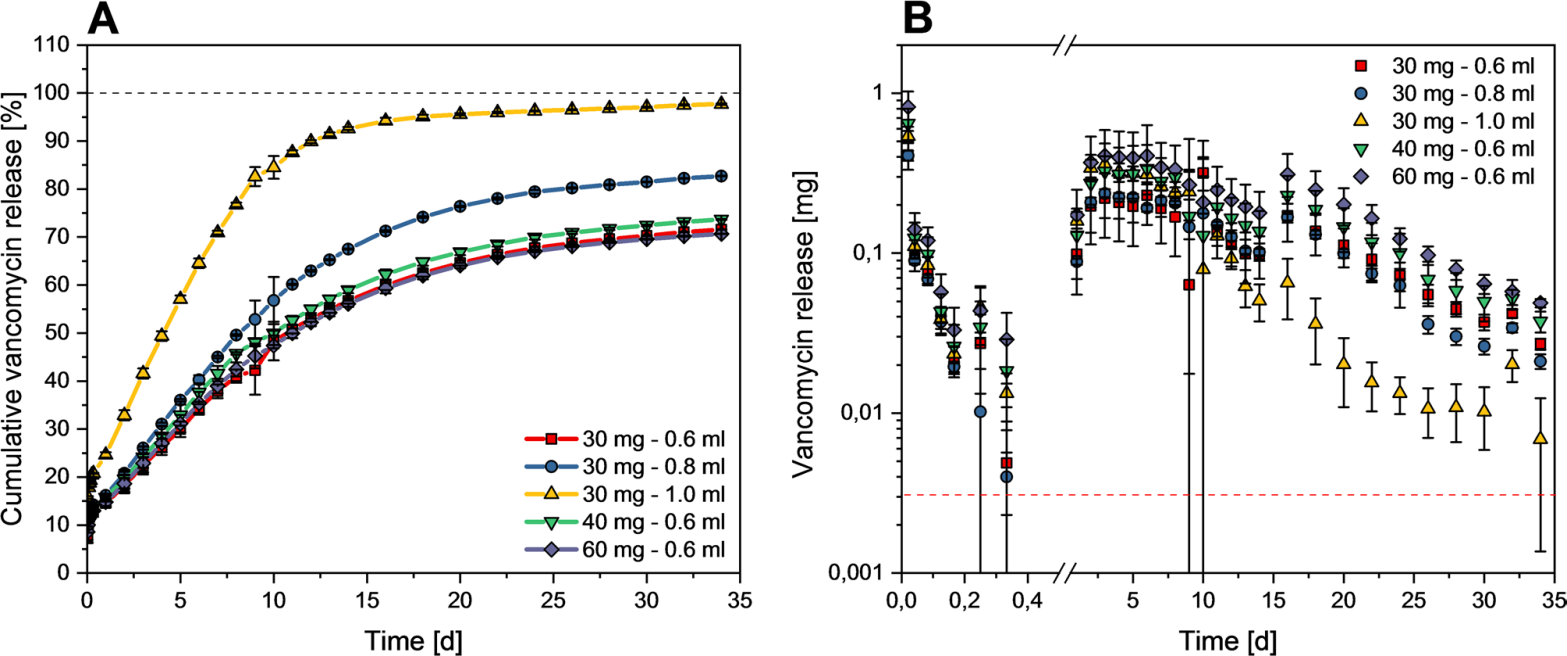
**(A)** Cumulative vancomycin release over 34 days from cement samples prepared by mixing 3.7 g ready-to-use cement paste with 0.6 ml vancomycin solution containing 30, 40 or 60 mg drug or by mixing the cement paste with either 0.6 ml, 0.8 ml or 1.0 ml solution containing 30 mg vancomycin. **(B)** Released vancomycin doses for each measurement time point. The red dotted line corresponds to the MIC of vancomycin against S. aureus [21]

**Fig. 7:**
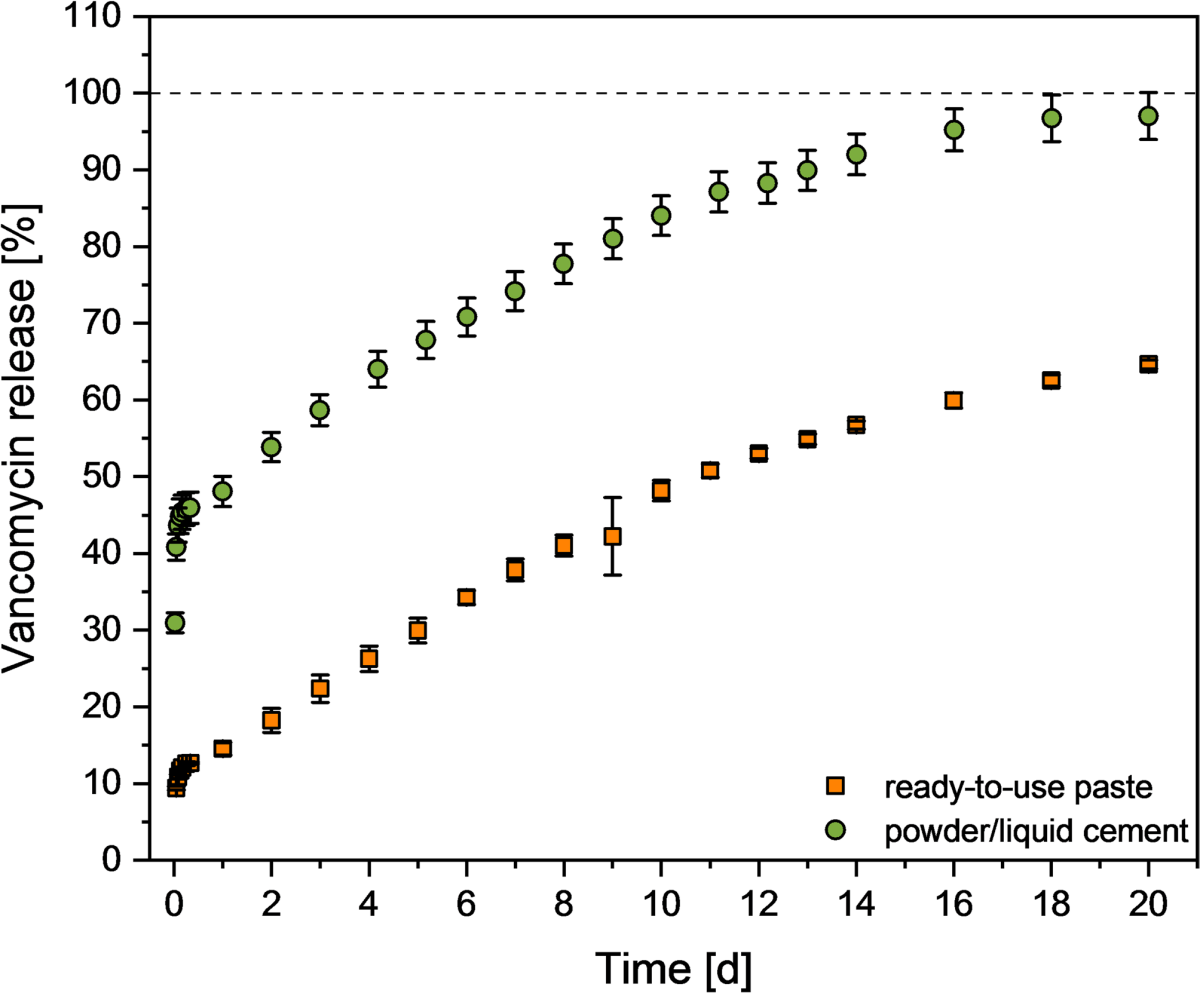
Comparison of vancomycin release profiles from ready-to-use cement paste (3.7 g cement paste mixed with 0.6 ml of 50 mg/ml vancomycin solution corresponding to 30 mg vancomycin in the paste) and a powder/liquid cement using a vancomycin solution with a concentration of 24.13 mg/ml at a PLR of 2.5 g/ml. Both pastes contained 6.98 mg vancomycin per 1 g hardened cement

The release profiles of vancomycin from the ready-to-use cement paste were characterized by a burst release of approx. 14.7–24.6% during the first 24 h, followed by typical first-order release kinetics (Fig. 6A). The release profile was similar for different vancomycin loadings (30, 40, and 60 mg) at a constant liquid volume of 0.6 ml with a total release of approx. 71–73% after 34 days. On the other hand, an increase in the amount of liquid with a constant vancomycin content (30 mg) accelerated the release with a total release of 83% (0.8 ml) and 98% (1.0 ml) after 34 days. Vancomycin doses released from all mixtures were found to exceed the MIC for vancomycin against S. aureus over the entire test period (Fig. 6B). When the release profile is compared to an aqueous powder/liquid cement paste with identical drug loading, the powder/liquid cement showed both a higher burst release during the first 24 h as well as a higher total release after 20 days (Fig. 7).

**Fig. 8:**
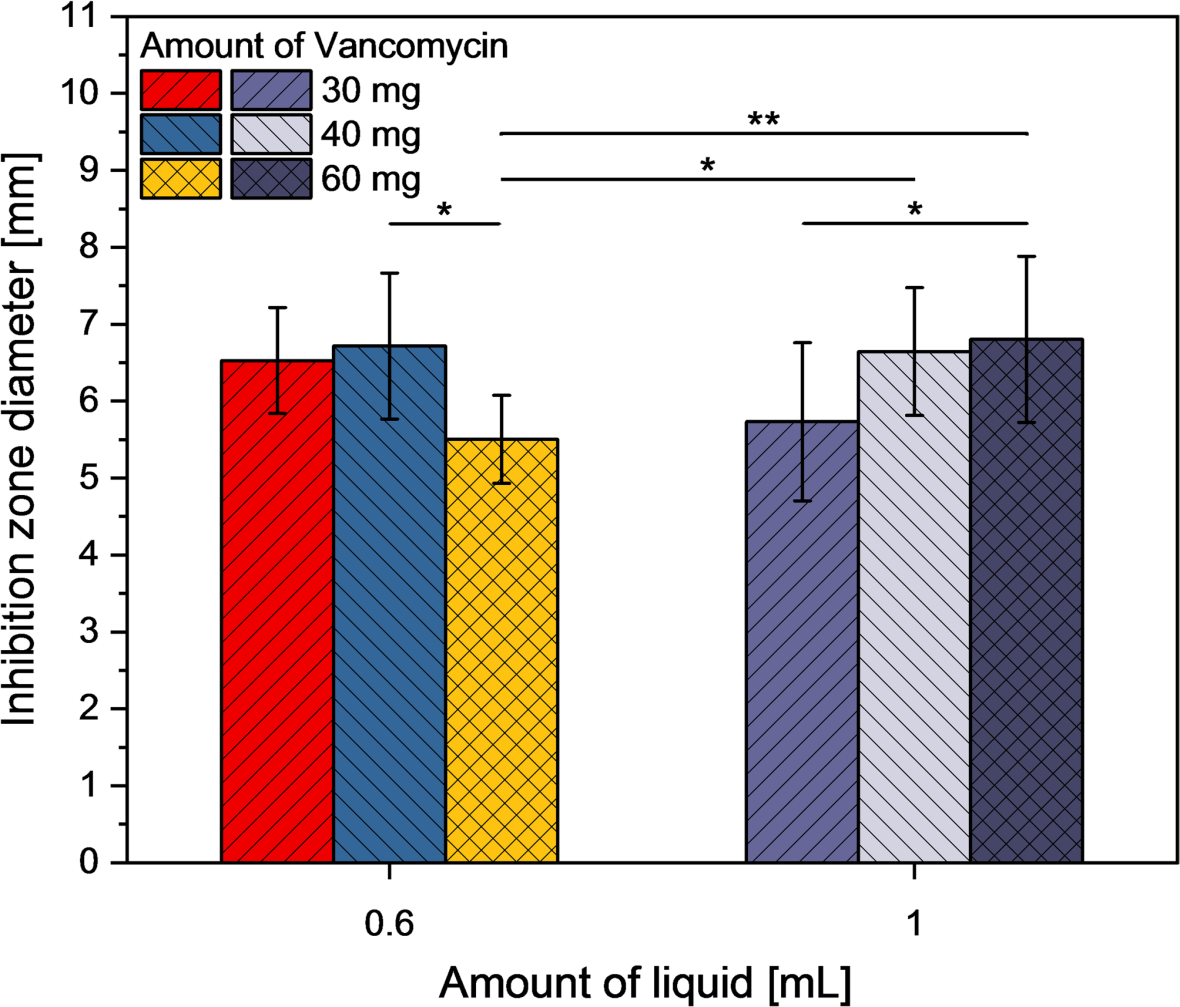
Inhibition zones around samples from ready-to-use pastes, prepared with different amounts of solution and vancomycin content, after 24 h at 37 °C against S. aureus. (*p < 0.05, *p < 0.01)

Finally, we demonstrated that the vancomycin-loaded ready-to-use pastes retained antimicrobial activity against S. aureus, as evidenced by inhibition zones measuring 5.4–6.8 mm around the cement samples in an agar diffusion test regime (Fig. 8).

## Discussion

Ready-to-use cement pastes, made of a calcium phosphate powder dispersed in an inert organic solvent, possess major advantages in clinical use compared to traditional aqueous powder/liquid cement pastes. That is because the latter requires intraoperative mixing and offers only a very limited time frame for the application to the bone defect due to the ongoing setting reaction. In contrast, ready-to-use cement pastes do not need any mixing step in the surgical theatre. In addition, the application can be interrupted at any time since the setting process of the paste only starts after being in contact with the wet environment of the bone defect. Even though this is a clear benefit, challenges persist in utilizing ready-to-use pastes for drug delivery applications. Mixing classical cements with drugs is relatively straightforward, achieved by either adding drug powder to the cement or by dissolving the drug in the cement liquid. However, modifying ready-to-use cement pastes with drugs is more challenging as they usually have to be ejected from the syringe, followed by manually mixing with the solid drug and refilling into the syringe. This procedure was simplified in the current study by using an adapter between two syringes such that mixing with a drug solution was achieved by repeated ejection of the paste into the opposite syringe. It was proven that repeating this step ten times resulted in macroscopically homogeneous pastes when mixing 0.6–1 ml vancomycin solution with 3.7 g cement paste. It is important to note, that this mixing process initiates the setting reaction of the ready-to-use cement paste and thus limits the time available for applying the (antibiotic-loaded) paste to a defect. However, by using different volumes of antibiotic solution it was possible to increase the setting time from 5 to 6 min for 0.6 ml liquid to 14 min for 1 ml liquid (Fig. 3). Thus, the cement system allows for adjusting the setting time after mixing and prolonging the application time without compromising the mechanical stability of the resulting cement (Fig. 5A). Although the advantage of an unlimited application time is lost after mixing with an aqueous solution, the setting times are in the range of normal powder/liquid cement (6.3 ± 0.6 min). Here, a clear advantage of the ready-to-use cement pastes is the much easier and safer preparation regime as mixing is performed in closed syringes instead of using open systems such as a mixing bowl or glass slab. In general, drug modification with vancomycin had no other adverse effects on the cement properties in terms of the mechanical strength (Fig. 5), phase composition (Fig. 2), or porosity and pore size distribution (Fig. 4).

The release of the drug from the cements was then studied under static conditions and compared to a powder/liquid cement with an identical mineral composition. Here, it was observed that the ready-to-use pastes exhibited a lower initial burst release of 14.5% after 24 h compared to 47.9% for the powder/liquid cement (Fig. 7). This may be explained by the presence of the oil phase within the cement which retards vancomycin diffusion from the cement matrix. Eventually, the oil phase is exchanged by water as shown by Heinemann et al. [12]. In addition, compared to the prefabricated cement pastes, the powder/liquid cement showed, on average, a total porosity increased by approximately 5% points (Fig. 4), as well as a significantly more pronounced fraction of mesopores in the size range around 0.05 μm, which could also have contributed to a stronger burst release effect. This in turn caused the vancomycin release rates for the antibiotic-loaded ready-to-use pastes to slowly approach the observed release from the powder/liquid cement reference. At later time points, the initially exponential release kinetics slowed down for both cement systems resulting in a nearly quantitative release of > 90% vancomycin after 20 days for the powder/liquid cement compared to only 65% for the ready-to-use paste (Fig. 7). This is due to structural changes of the cement matrix during setting as also observed in other studies investigating the release kinetics from freshly mixed powder/liquid cement pastes [22–24]. Canal et al. [24] proposed that structural changes in the cement matrix during the setting process are the reason for the time-dependent antibiotic release. At the beginning of the setting process, the cement paste can still be considered as a suspension of mainly α-tricalcium phosphate particles in a liquid, from which nanosized hydroxyapatite crystals precipitate during the further course of cement hardening. This precipitation increases the tortuosity of the matrix and thereby slows down the release of the added drug. The cements tested in the present study were set under the same conditions at 100% humidity and 37 °C for 1 h before the start of the release tests, making them comparable with the tests conducted in the work of Canal et al. Since the cements still contained approx. 30% α-TCP even after 7 days (Fig. 2) and the conversion of the mineral phases to hydroxyapatite was therefore time dependent, the hypothesis proposed by Canal et al. [24] could also be an explanation for the release profiles observed in the present study.

Furthermore, the released amount of antibiotic at each time point linearly increased when its amount added at a constant liquid volume of 0.6 ml per 3.7 g cement paste was increased from 30 mg to 60 mg (Fig. 6B). Therefore, no influence on cumulative release kinetics was observed at a constant liquid volume (Fig. 6A). In contrast, increasing the volume of vancomycin solution accelerated the drug release kinetics, resulting in a quantitative release when using 1 ml of solution after 34 days. This can be explained by changes in the total porosity and pore size distribution, since a close correlation exists between the release rates of the antibiotic from the cements and their porosity [24, 25]. Even though no considerable difference in the overall porosity could be observed in the samples analysed, there are still fine structural differences. With increasing amounts of vancomycin solution introduced, the global maximum shifts towards larger pore sizes (Fig. 4). This explains the faster release from the cements mixed with higher volumes of vancomycin solution, as they contain larger pores. In addition, the total porosity of the antibiotic-loaded ready-to-use cements of the present study is comparable to similar cements produced from oil-based mineral pastes, reaching a maximum of nearly 30% [19].

It should be noted that drug release kinetics in vivo may differ significantly from the profiles observed here due to varying environmental conditions. While the results presented here require validation through clinical trials and/or preclinical in vivo studies, they nonetheless provide an initial assessment of the suitability of the materials and methods for clinical application and form a foundation for further investigations of other cement systems with different active agents.

According to Pastorino et al. [26] and Geurts et al. [27], effective local antibiotic therapy requires local drug concentrations that are significantly above the minimal inhibitory concentration (MIC) of the corresponding microorganisms in the first few days, followed by a gradual release of the drug at a lower level over several weeks. Such a release pattern was also observed in the experiments carried out in the current study. The cut-off value of the MIC for methicillin-sensitive Staphylococcus aureus (MSSA) and methicillin-resistant Staphylococcus aureus (MRSA) is 2 µg/ml [28, 29]. In all analysed sample supernatants, the concentration of the active substance was significantly above this value over the entire observation period of 34 days demonstrating the antimicrobial potential of the antibiotic-loaded ready-to-use cement pastes. Furthermore, we could demonstrate by an agar diffusion test that the released vancomycin still had a high antimicrobial activity against S. aureus (Fig. 8). In summary, this shows that the antibiotic-loaded cements have the potential for effective local antibiotic therapy by enabling high biologically relevant antibiotic concentrations over several weeks.

## Conclusion

The present study describes the modification of an oil-based, ready-to-use calcium phosphate bone cement paste with an antibiotic solution. The two-syringe setup used offers an easy and fast way to homogeneously mix both components resulting in a directly applicable cement paste. Using vancomycin as a model antibiotic, we were able to demonstrate the effectiveness of such cement modification, since the release rates can be controlled by the amount and concentration of antibiotic liquid. Furthermore, the antibiotic-loaded ready-to-use cement pastes showed advantages over powder/liquid cements such as a reduced burst release and subsequent prolonged antibiotic release. The released doses exceeded the minimum inhibitory concentration for S. aureus over more than 30 days. Furthermore, the drug modification had no adverse effect on other material properties of the cements such as mechanical strength or mineral phase composition after setting. Clinical applications of the presented drug-eluting ready-to-use CPC paste might be both the local treatment of an existing osteomyelitis or the prevention of a post-operative bone infection. Compared to conventional treatment options, like drug-loaded spacers made from PMMA, the antibiotic-modified CPC paste has the advantage that it not only offers the release of antimicrobial substances but simultaneously serves as a resorbable bone void filler. This eliminates the need for implant removal, as it is necessary for non-degradable polymeric drug delivery devices. Although the ready-to-use cement pastes showed significantly lower strengths compared to a conventional powder/liquid cement of similar mineral composition, they offer application-relevant advantages such as efficient and error-resistant mixing, along with excellent storage stability of the pre-mixed mineral starting components.

## Data Availability

Data is provided within the manuscript or supplementary information files.
